# The Effect of Allicin on the Proteome of SARS-CoV-2 Infected Calu-3 Cells

**DOI:** 10.3389/fmicb.2021.746795

**Published:** 2021-10-28

**Authors:** Kirstin Mösbauer, Verena Nadin Fritsch, Lorenz Adrian, Jörg Bernhardt, Martin Clemens Horst Gruhlke, Alan John Slusarenko, Daniela Niemeyer, Haike Antelmann

**Affiliations:** ^1^Institute of Virology, Berlin Institute of Health, Charité-Universitätsmedizin Berlin, Freie Universität Berlin, Berlin, Germany; ^2^German Centre for Infection Research (DZIF), Berlin, Germany; ^3^Institute for Biology-Microbiology, Freie Universität Berlin, Berlin, Germany; ^4^Department Environmental Biotechnology, Helmholtz Centre for Environmental Research-UFZ, Leipzig, Germany; ^5^Fachgebiet Geobiotechnologie, Technische Universität Berlin, Berlin, Germany; ^6^Institute for Microbiology, University of Greifswald, Greifswald, Germany; ^7^Department of Plant Physiology, RWTH Aachen University, Aachen, Germany

**Keywords:** allicin, SARS-CoV-2, proteome, Vero E6, Calu-3

## Abstract

Allicin (diallyl thiosulfinate) is the major thiol-reactive organosulfur compound produced by garlic plants (*Allium sativum*) upon tissue damage. Allicin exerts its strong antimicrobial activity against bacteria and fungi via *S*-thioallylation of protein thiols and low molecular weight thiols. Here, we investigated the effect of allicin on SARS-CoV-2 infected Vero E6 and Calu-3 cells. Toxicity tests revealed that Calu-3 cells showed greater allicin tolerance, probably due to >4-fold higher GSH levels compared to the very sensitive Vero E6 cells. Exposure of infected Vero E6 and Calu-3 cells to biocompatible allicin doses led to a ∼60–70% decrease of viral RNA and infectious viral particles. Label-free quantitative proteomics was used to investigate the changes in the Calu-3 proteome after SARS-CoV-2 infection and the effect of allicin on the host-virus proteome. SARS-CoV-2 infection of Calu-3 cells caused a strong induction of the antiviral interferon-stimulated gene (ISG) signature, including several antiviral effectors, such as cGAS, Mx1, IFIT, IFIH, IFI16, IFI44, OAS, and ISG15, pathways of vesicular transport, tight junctions (KIF5A/B/C, OSBPL2, CLTCL1, and ARHGAP17) and ubiquitin modification (UBE2L3/5), as well as reprogramming of host metabolism, transcription and translation. Allicin treatment of infected Calu-3 cells reduced the expression of IFN signaling pathways and ISG effectors and reverted several host pathways to levels of uninfected cells. Allicin further reduced the abundance of the structural viral proteins N, M, S and ORF3 in the host-virus proteome. In conclusion, our data demonstrate the antiviral and immunomodulatory activity of biocompatible doses of allicin in SARS-CoV-2-infected cell cultures. Future drug research should be directed to exploit the thiol-reactivity of allicin derivatives with increased stability and lower human cell toxicity as antiviral lead compounds.

## Introduction

The Severe Acute Respiratory Syndrome Coronavirus 2 (SARS-CoV-2) causes Coronavirus disease (COVID-19), which represents a global health burden (Zhou et al., 2020). COVID-19 is often associated with immunopathology since severely ill patients had decreased levels of T lymphocytes, including regulatory T cells, cytotoxic and helper T cells, and natural killer cells (Qin et al., 2020; Wei et al., 2020). Patients with severe illness showed a cytokine storm syndrome associated with a dysregulated immune activation and hyperinflammation (Fara et al., 2020). High levels of pro-inflammatory cytokines IL-1ß, IL-2, IL-6, IL-7, IL-10, macrophage inflammatory protein-1A (MIP-1A), TNF-α, and INF-γ have been detected, connecting the uncontrolled inflammation and dysregulation of the immune response with the high mortality in severely ill COVID-19 patients (Fara et al., 2020; Qin et al., 2020; Wei et al., 2020). While mild infections were characterized by highly activated HLA-DR^*hi*^CD11c^*hi*^ inflammatory monocytes with the interferon-stimulated gene (ISG) signature, severe illness was manifested by dysfunctional neutrophil precursors, and HLA-DR^*lo*^ monocytes with pro-inflammatory functions (Schulte-Schrepping et al., 2020). These immunological markers of pro-inflammatory cytokines and the dysfunctional myeloid compartment might help to identify drug targets to prevent progression to severe illness (Fara et al., 2020; Schulte-Schrepping et al., 2020).

While global vaccination campaigns are underway, the development of efficient therapies to prevent COVID-19 disease progression is an urgent need. Garlic plants (*Allium sativum*) produce volatile organosulfur compounds, such as diallyl thiosulfinate (allicin) and diallyl polysulfanes, which are known for their antimicrobial, antiviral, anticancer, anti-inflammatory and immunomodulatory effects (Borlinghaus et al., 2014, 2021; Schäfer and Kaschula, 2014). Garlic compounds showed broad-spectrum antimicrobial activity against several pathogenic bacteria, viruses, fungi, and parasites (Rabinkov et al., 1998; Münchberg et al., 2007; Block, 2010; Borlinghaus et al., 2014, 2021; Reiter et al., 2017; Arbach et al., 2019; Loi et al., 2019; Rouf et al., 2020).

Allicin is a thiol-reactive compound, which reacts with Cys thiols via thiol-disulfide exchange reactions, leading to *S*-thioallylations of proteins (Miron et al., 2000, 2010). Widespread *S*-thioallylations of redox-sensitive Cys residues in proteins were identified in the proteome of human Jurkat cells, *Escherichia coli*, *Staphylococcus aureus*, and *Bacillus subtilis* (Rabinkov et al., 1998; Miron et al., 2010; Müller et al., 2016; Chi et al., 2019; Gruhlke et al., 2019; Loi et al., 2019). In Jurkat cancer cells, 332 *S*-thioallylated proteins were identified 10 min after allicin treatment, including highly abundant cytoskeleton proteins, HSP90 chaperones, translation elongation factors and glycolytic enzymes. Allicin caused disruption of the actin cytoskeleton, enzymatic inactivation and Zn^2+^ release to stimulate the IL-1-dependent IL-2 secretion by T cells as an immunomodulatory effect (Gruhlke et al., 2019).

In addition, *S*-thioallylations deplete low molecular weight thiols, such as glutathione (GSH) and bacillithiol (BSH) in bacteria and yeast cells (Gruhlke et al., 2010, 2019; Arbach et al., 2019). Thus, allicin leads to oxidative stress responses, inhibition of protein functions and an impaired cellular redox balance. Since SARS-CoV-2 is rich in Cys residues in its surface spike glycoprotein, a reduced state of the host cell cytoplasm is required for efficient virus entry and membrane fusion. Moreover, allicin is cell permeable and has been shown to cause transient pore formation in phospholipid membranes (Miron et al., 2000; Gruhlke et al., 2015). The antiviral effect of allicin has been previously investigated against several respiratory viruses, including influenza, SARS-CoV and rhinovirus (Rouf et al., 2020).

In this work, we show that allicin at biocompatible doses decreases infectious viral particles and viral RNA of SARS-CoV-2 in the primate kidney-derived cell line Vero E6 and the human lung cell line Calu-3. We further identified proteome changes caused by SARS-CoV-2 infection and the effect of allicin on these host pathways. While the interferon-stimulated gene (ISG) signature was most prominently upregulated in SARS-CoV-2 infected Calu-3 cells, the ISG response and several host cellular pathways were restored to levels of untreated cells by allicin. Thus, allicin exerts an antiviral and immunomodulatory effect when applied in infected cell cultures *in vitro*, which is supported at the proteome level.

## Materials and Methods

### Cultivation of Cell Lines and Infection Experiments With SARS-CoV-2

Vero E6 (ATCC CRL-1586) and Calu-3 (ATCC HTB-55) cell lines were cultivated in Dulbecco’s Modified Eagle’s Medium (DMEM), supplemented with 10% fetal bovine serum (FBS), 1% non-essential amino acids and 1% sodium pyruvate (Gibco), and grown at 37°C and 5% CO_2_. Cell lines were free of mycoplasma, authenticated based on morphology and growth properties and confirmed by PCR. The cell cultures were used for viability or infection assays below cell passage 20. No antibiotics have been used during cultivation of eukaryotic cells.

The infection experiments were performed with SARS-CoV-2 Munich isolate (CSpecVir985) under biosafety level 3 conditions with appropriate respiratory personal protection equipment. Vero E6 and Calu-3 cells were seeded at densities of 3.5 × 10^5^ or 6 × 10^5^ cells/ml in 12-well TC plates (TPP Techno Plastic Products AG), respectively. After 24 h, cells were infected at a MOI of 0.01 or 0.005, diluted in serum-free OptiPro medium for 1 h at 37°C. The medium was removed and cells were washed twice with phosphate-buffered saline (PBS) followed by addition of DMEM and supplements. Samples were taken at 16 and 24 h p.i. for further analysis.

### Allicin Synthesis and Treatment

Allicin was synthesized by oxidation of 3-[(Prop-2-en-1-yl)disulfanyl]prop-1-ene (diallyl disulfide, Sigma-Aldrich, Germany) with peracetic-acid (glacial acetic acid/H_2_O_2_) as described previously (Gruhlke et al., 2010). To analyze the antiviral effect of allicin, SARS-CoV-2 infection experiments were performed with an allicin pre- and post-treatment of Vero E6 cells. For the pre-treatment, either the cells or the virus dilution were incubated with 50 μM allicin for 30 min. We have chosen 50 μM allicin since this concentration was determined as sub-lethal for Vero E6 cells. Pre-treated cells were washed with PBS and infected according to the infection protocol as described above. Pre-treated virus was used in the infection experiment according to the protocol above. Post-treatment of SARS-CoV-2 infected cells was accomplished by adding the indicated concentration of allicin into the medium after infection. Thus, in the post-treatment protocol, the added allicin remained on the infected cells until sample collection after 16 and 24 h.

### Cell Viability Assay

The cell viability of Vero E6 and Calu-3 cells was analyzed by quantification of ATP levels using the CellTiter-Glo^®^ Luminescent Cell Viability Assay (Promega) according to the instructions of the manufacturer. The cells were cultivated as described above in 96-well flat clear bottom black TC-treated microplates (Corning^®^) and exposed to different amounts of allicin for 24 h. Cell viability of treated cells was normalized to non-treated cells.

### Determination of the Levels of Glutathione and Glutathione Disulfide in Vero E6 and Calu-3 Cells

Vero E6 and Calu-3 cells were cultivated as described above in 96-well white opaque flat bottom tissue culture plate (Falcon) and seeded at densities of 1 × 10^4^ cells/well. After washing with PBS, the intracellular GSH and glutathione disulfide (GSSG) concentrations were determined using the GSH/GSSG-Glo^TM^ assay (Promega) according to the instructions of the manufacturer for adherent cells. Briefly, total GSH levels were measured in one sample by reduction of GSSG to GSH using DTT. Total GSSG amounts were measured in a second sample by blocking reduced GSH with *N*-ethylmaleimide (NEM), followed by GSSG reduction with DTT. The GSH transferase (GST) uses GSH as cofactor to convert luciferin-NT to GSH-NT resulting in the release of luciferin. Luciferin is oxidized to oxyluciferin by the Ultra-Glo^TM^ rLuciferase, leading to emission of chemiluminescence, which was measured using an integration time of 1 s/well by the CLARIOstar microplate reader (BMG Labtech). GSH levels were calculated based on GSH standard curves. For determination of the cellular GSH levels, the GSSG amounts were subtracted from the total GSH level.

### Plaque Titration Assay

The number of infectious virus particles was determined by a plaque titration assay. Vero E6 monolayers were seeded in 24-well TC plates (TPP Techno Plastic Products AG) and infected with 200 μl of serial dilutions of SARS-CoV-2 containing cell culture supernatants of infected Vero E6 or Calu-3 cells, which were diluted in OptiPro serum-free medium. After 1 h adsorption, the supernatant was removed and cells overlaid with 1.2% Avicel (FMC BioPolymers) diluted in DMEM. After 72 h, the overlay was removed, cells were fixed in 6% formaldehyde and plaques were visualized by crystal violet staining.

### Viral RNA Extraction and Real-Time Reverse-Transcription PCR

Viral RNA extraction was performed from 50 μl culture supernatant of SARS-CoV-2 infected Vero E6 and Calu-3 cells using the viral RNA kit (Macherey-Nagel) according to the instructions of the manufacturer. SARS-CoV-2 genome equivalents (GE) were detected by quantitative RT–PCR [LightCycler 480 Real-Time PCR System and Software version 1.5 (Roche)], targeting the SARS-CoV-2 *E* gene using the primers *E* gene-F (5′-ACAGGTACGTTAATAGTTAATAGCGT-3′) and *E* gene-R (5′-ATATTGCAGCAGTACGCACACA-3′). Absolute quantification was performed using SARS-CoV-2 specific *in vitro*-transcribed RNA standards as described previously (Corman et al., 2020).

### Proteome Analysis of SARS-CoV-2 Infected Host Cells Using Orbitrap Fusion Mass Spectrometry

6 × 10^5^ Calu-3 cells per sample were infected with SARS-CoV-2 as described above and treated with 150 μM allicin for 24 h. Calu-3 cells were harvested by centrifugation. The cell pellets were washed with PBS and alkylated for 15 min at room temperature (RT) under denaturing conditions in 200 μl of UCE-IAM buffer, consisting of 8 M urea, 1% (w/v) CHAPS, 1 mM EDTA, 200 mM Tris–HCl pH 8.0 and 100 mM IAM as described (Rossius et al., 2018). Subsequently, the alkylated protein extracts were precipitated with trizol and 96% ethanol and washed four times with 1 ml 70% ethanol. The protein pellets were separated by a short 15% non-reducing SDS-PAGE, which was running for 15 min and stained with Colloidal Coomassie Blue. The gel fractions were cut and in-gel tryptic digested as described previously (Rossius et al., 2018). The eluted peptides were desalted using ZipTip-μC18 material (Merck Millipore) and dissolved in 0.1% (v/v) formic acid before LC-MS/MS analysis. The peptide samples of non-infected Calu-3 cells (Mock) and SARS-CoV-2 infected Calu-3 cells with and without allicin treatment were subjected to nLC-MS/MS analysis using an Orbitrap Fusion (Thermo Fisher Scientific) coupled to a TriVersa NanoMate (Advion, Ltd.) as described previously (Kublik et al., 2016). Peptide identification of the human and SARS-CoV-2 proteome was performed by Proteome Discoverer (version 2.2, Thermo Fisher Scientific) using the SequestHT search engine as described (Seidel et al., 2018). Human and SARS-CoV-2 proteins were identified by searching all tandem MS/MS spectra against the human proteome protein sequence database (20,286 entries) extracted from UniprotKB release 12.7 (UniProt Consortium, Nucleic acids research 2007, 35, D193-197) as well as against the European Virus Archive Global # 026V-03883 sequence database. Peptides were considered to be identified with high confidence at a target false discovery rate of ≤0.01 and with a medium confidence at ≤0.05, based on the *q*-values. Identified proteins were quantified by the “Percursor Ions Quantifier” implemented in Proteome Discoverer 2.2 based on peak intensities to estimate the abundance of the human and SARS-CoV-2 proteins in the peptide samples. Error tolerance for precursor ion and fragment ion *m/z* values was set to 3 ppm and 0.5 Da, respectively. Two missed cleavage sites were allowed. Methionine oxidation (+15.994915 Da), cysteine carbamidomethylation (+57.021464 Da) and cysteine *S*-thioallylation by allicin (+72.00337 Da for C_3_H_5_S_1_) were set as variable modifications. The mass spectrometry data have been deposited to the ProteomeXchange Consortium via the PRIDE partner repository (Perez-Riverol et al., 2019; Deutsch et al., 2020) with the dataset identifier PXD024375.

### Statistical Analyses

Statistical analysis of the cell viability assays, the GSH and GSSG measurements as well as the determination of viral RNA and infectious particles were performed from 3–4 biological replicates with 1–3 technical replicates using the Student’s unpaired two-tailed *t*-test for two samples with unequal variance. Proteomics analyses were performed from 3–4 biological replicates with 1–3 technical replicates. For calculation of the statistics of the proteomics data, the LFQ intensity values of each proteomics sample and every single treatment were tested for normality by using Jarque Bera (testing for kurtosis and skewness) (Jarque and Bera, 1980) and Anderson Darling (based on Kolmogorov-Smirnov) tests (Anderson and Darling, 1954). Accordingly, *p*-values for pairwise treatment comparisons were calculated by the Welsh test (Student’s unpaired two-tailed *t*-test for two samples with unequal variance and heteroscedastic data). The *p*-values and significance levels are included in the figure and table legends.

## Results

### Biocompatible Allicin Concentrations Correlate With the Intracellular Glutathione Levels in Vero E6 and Calu-3 Cells

**FIGURE 1:**
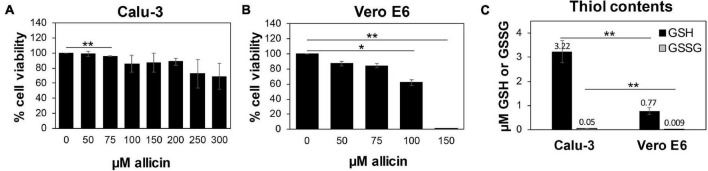
Human Calu-3 cells are more resistant to allicin compared to Vero E6. **(A,B)** Cell viability of untreated and allicin treated Calu-3 **(A)** and Vero E6 cells **(B)** was measured after 24 h using the CellTiter-Glo^®^ Luminescent Cell Viability Assay (Promega) according to the manufacturer’s instructions. The viability of the control without allicin was set to 100%. The viability of Calu-3 cells was not significantly decreased upon exposure to 100–200 μM allicin, while concentrations of ≥100 μM allicin interfered with Vero E6 cell viability. **(C)** The levels of glutathione (GSH) and glutathione disulfide (GSSG) were determined in untreated Calu-3 and Vero E6 cells using the GSH/GSSG-Glo Assay (Promega) according to the manufacturer’s instructions. The results are from 4 biological replicates with 3 technical replicates for **(C)**. Error bars represent the standard deviation (SD). *p*-values were calculated using an unpaired two-tailed *t*-test.*p < 0.05; **p < 0.01.

While allicin has many beneficial effects for human health, crushed garlic is also toxic and harmful for human cells. Fresh garlic can cause severe cellular and tissue damage upon direct exposure to the epithelial cells and mucous membranes of the respiratory tract and the skin, such as garlic burns (Bautista et al., 2005; Al-Qattan, 2009; Vargo et al., 2017; Hitl et al., 2021; Muniz et al., 2021). Thus, we first assessed the toxicity of allicin in Calu-3 and Vero E6 cells, which are used here as cell culture models for SARS-CoV-2 infection. Using cell viability assays, the biocompatible, non-harmful doses of allicin in Calu-3 and Vero E6 cells were determined. Both cell lines differed strongly in their susceptibilities toward allicin. Calu-3 cells showed high viability rates of ∼85% after treatment with 200 μM allicin. Even concentrations of 300 μM allicin decreased the viability rate of Calu-3 cells only non-significantly to ∼70% (Figure 1A). Treatment of Vero E6 cells with 75 μM allicin led to a cell viability rate of 84% (Figure 1B), whereas 150 μM allicin resulted in killing of 99% of Vero E6 cells. Thus, the sub-lethal biocompatible doses of allicin were determined as 50–75 μM in Vero E6 cells and 100–200 μM in the more tolerant Calu-3 cells.

Previous studies already revealed strong variations in the susceptibilities of different cell lines toward allicin, which correlated with different intracellular GSH contents (Gruhlke et al., 2016, 2019). Thus, we measured the intracellular GSH and GSSG levels in Vero E6 and Calu-3 cells (Figure 1C). The GSH content of the more tolerant Calu-3 cells was determined as 3.2 μM, which was 4.2-fold higher compared to only 0.77 μM GSH as measured in Vero E6. As expected, the amounts of GSSG were very low with 0.05 μM and 0.009 μM in Calu-3 and Vero E6 cells, respectively. These data suggest that Calu-3 cells show greater allicin tolerance in part due to their higher GSH levels compared to Vero E6 cells.

### Allicin Leads to Decreased Infectious Viral Particles and Viral RNA in SARS-CoV-2 Infected Vero E6 and Calu-3 Cells

**FIGURE 2:**
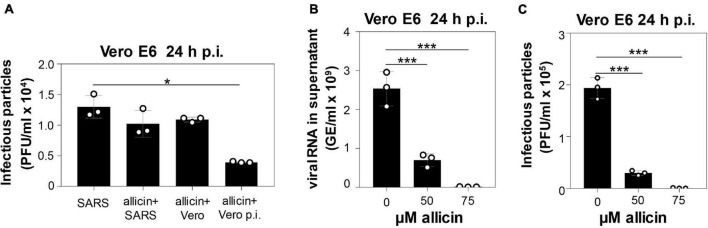
Allicin treatment of SARS-CoV-2 infected Vero E6 cells reduced the levels of infectious viral particles and viral RNA. **(A–C)** Vero E6 cells were infected with SARS-CoV-2 at a MOI of 0.01. After 24 h p.i. viral replication was analyzed by determination of infectious viral particles or viral RNA in the supernatant. **(A)** Comparison of different allicin treatments: Untreated Vero E6 cells infected with SARS-CoV-2 as control (SARS); SARS-CoV-2 pre-treated with 50 μM allicin for 30 min prior to infection of host cells (allicin + SARS); host cells pre-treated with 50 μM allicin for 30 min prior to infection with SARS-CoV-2 (allicin + Vero); and SARS-CoV-2 infected host cells treated with 50 μM allicin p.i. (allicin + Vero p.i.). **(B,C)** The amount of viral RNA **(B)** and infectious viral particles **(C)** was determined after treatment of SARS-CoV-2 infected Vero E6 cells with 50 and 75 μM allicin p.i. The results **(A–C)** are from three biological replicates with two technical replicates for panel **(B)**. Error bars represent the SD. *p*-values were calculated using an unpaired two-tailed *t*-test.*p < 0.05; ***p < 0.001.

The antiviral effect of allicin against SARS-CoV-2 was analyzed using pre- and post-treatment options for the more allicin-sensitive infected Vero E6 cells: (1) Cells were pre-exposed to 50 μM allicin for 30 min before SARS-CoV-2 infection. (2) The virus was treated with 50 μM allicin for 30 min prior to infection. (3) SARS-CoV-2 infected Vero E6 cells were treated with 50 μM allicin post infection (p.i.) (Figure 2A). We have chosen 50 μM allicin since this concentration did not affect viability of Vero E6 cells (Figure 1B). The number of infectious SARS-CoV-2 particles (PFU, plaque forming units) was determined 24 h p.i. by the plaque titration assay. However, only post-treatment with 50 μM allicin led to a significant 70% decrease in the amount of infectious virus particles, whereas the pre-treatment of cells or virus caused only a 16–21% reduction of viral plaques (Figure 2A). These results suggest that allicin might affect host-virus interactions by its antiviral and immunomodulatory activities.

In addition, viral RNA genome equivalents (GE) were determined from the supernatant of infected Vero E6 cells using quantitative RT-PCR. In agreement with the plaque assays, the qRT-PCR results revealed a 72% lower amount of viral RNA after addition of 50 μM allicin to SARS-CoV-2 infected Vero E6 cells (Figures 2B,C). Moreover, virus plaque assays and qRT-PCR results showed an almost complete >99% inhibition of SARS-CoV-2 replication after exposure to 75 μM allicin, supporting the strong antiviral activity of allicin in infected Vero E6 cells (Figures 2B,C).

**FIGURE 3:**
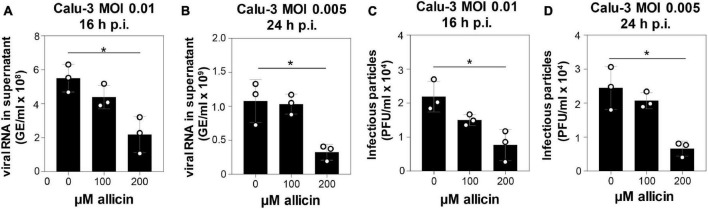
Allicin treatment of SARS-CoV-2 infected Calu-3 cells leads to decreased amounts of infectious viral particles and viral RNA. **(A–D)** SARS-CoV-2 infected Calu-3 cells were treated with 100 and 200 μM allicin p.i. The amount of viral RNA **(A,B)** and infectious viral particles **(C,D)** was determined 16 h **(A,C)** and 24 h **(B,D)** p.i. with SARS-CoV-2 at a MOI of 0.01 **(A,C)** and 0.005 **(B,D)**. The results are from three biological replicates with two technical replicates for panels **(A,B)**. Error bars represent the SD. *p*-values were calculated using an unpaired two-tailed *t*-test. *p < 0.05.

The antiviral effects of biocompatible doses of allicin were further analyzed in the more allicin-resistant Calu-3 cells. After infection with SARS-CoV-2 at a multiplicity of infection (MOI) of 0.01 and 0.005, Calu-3 cells were treated with biocompatible doses of 100 and 200 μM allicin and analyzed 16 and 24 h p.i., respectively (Figure 3). Treatment of infected Calu-3 cells with 100 μM allicin did not significantly inhibit viral replication (Figures 3A–D). However, exposure of infected Calu-3 cells to 200 μM allicin led to a significant >60% decrease of viral RNA (Figures 3A,B) and a >65% reduction of infectious particles (Figures 3C,D).

**FIGURE 4:**
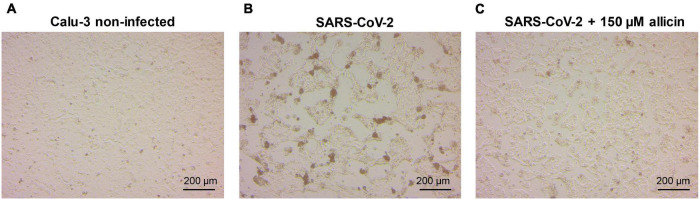
Cellular effect of 150 μM allicin on SARS-CoV-2 infected Calu-3 cells. Non-infected Calu-3 cells were cultivated for 24 h and served as controls **(A)**. Calu-3 cells were infected with SARS-CoV-2 at a MOI of 0.01 and the SARS-CoV-2 induced cellular effects were studied at 24 h p.i. without **(B)** or with 150 μM allicin treatment **(C)** after infection as described in the “Materials and Methods” section. At 24 h p.i. infected Calu-3 cells showed cellular damages, including cell rounding, detachment and cell death **(B)**. Allicin treatment decreased some cellular damages **(C)**. Cells were imaged with a Nikon Ts2R-FL inverted microscope.

The antiviral effect of allicin on SARS-CoV-2 infected Calu-3 cells was further supported by microscopy imaging (Figures 4A–C). While SARS-CoV-2 infection at a MOI of 0.01 resulted in cellular damage of Calu-3 cells after 24 h p.i., the addition of 150 μM allicin partially protected the cells against this damage (Figures 4B,C). Taken together, our results indicate that biocompatible allicin doses exert an antiviral effect against SARS-CoV-2 in both Vero E6 and Calu-3 cells.

### Changes in the Calu-3 Proteome After SARS-CoV-2 Infection

**FIGURE 5:**
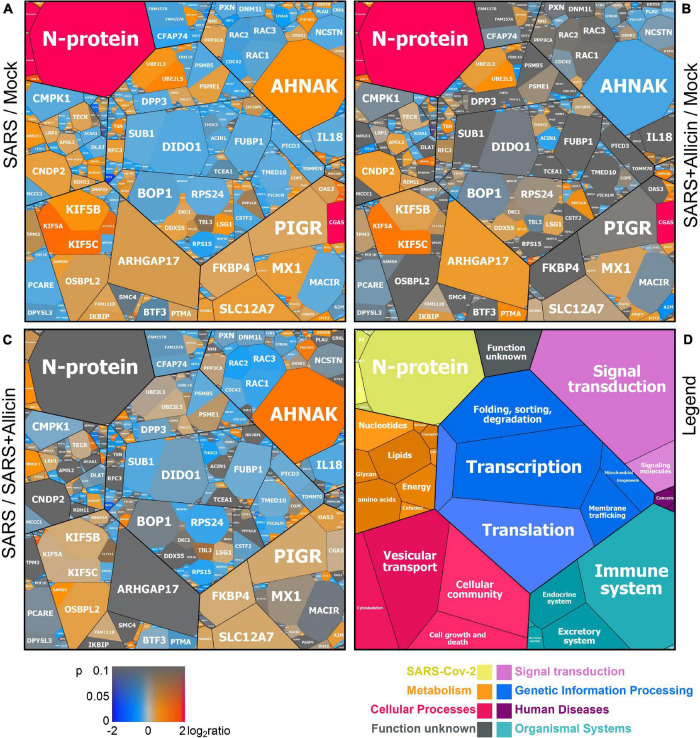
The effect of allicin on the proteome of SARS-CoV-2 infected Calu-3 cells. The host-viral proteome treemaps **(A–C)** show the 536 differentially expressed proteins upon SARS-CoV-2 infection and were constructed by the Paver software (Mehlan et al., 2013). The treemaps visualize the following proteome changes: **(A)** SARS-CoV-2 infection/Mock, **(B)** SARS-CoV-2 infection + Allicin/Mock and **(C)** SARS-CoV-2 infection −/+ Allicin. The treemap **(D)** serves as legend for the functional KEGG categories displayed in different colors for level 1 and sublevel 2 as listed in Supplementary Table 3. The cell sizes in panels **(A–C)** denote the average abundances of 207 proteins with ≥1.5-fold inductions and 329 proteins with <0.66-fold decreased expression after SARS-CoV-2 infection. The log2 ratios of the proteins are shown by a red-blue color gradient (red – induction, blue – repression) **(A–C)**. *p*-values (*p* < 0.05; *p* < 0.01) were calculated using an unpaired two-tailed *t*-test from 3 to 4 biological replicates with 1–3 technical replicates (Supplementary Table 3).

Label-free quantitative (LFQ) proteomics by Orbitrap Fusion LC-MS/MS analysis was used to investigate the changes in the proteome of Calu-3 cells after SARS-CoV-2 infection and the effect of 150 μM allicin. The concentration of 150 μM allicin was chosen since this was sub-lethal for Calu-3 cells (Figure 1A), and protected the cells against SARS-CoV-2 damage (Figure 4C). The proteome samples of Calu-3 cells were analyzed before infection (Mock) and 24 h p.i. with SARS-CoV-2 at a MOI of 0.01 in the absence or presence of 150 μM allicin in 3–4 biological and 1–3 technical replicates. The total LFQ intensities of all proteins in each sample were normalized and represent 100% of the total protein abundance. Overall, we quantified 4,251 proteins, including 4,243 Calu-3 host proteins and 8 SARS-CoV-2 proteins in the total proteome (Supplementary Tables 1, 2). After infection, about 207 and 329 proteins were ≥1.5-fold induced and <0.66-fold decreased, respectively (Supplementary Table 3). These 536 differentially expressed proteins contribute to only 2.73% of the total proteome abundance in SARS-CoV-2 infected Calu-3 cells (Supplementary Table 3). The proteins were sorted into KEGG Ontology (KO) or Uniprot categories and their fold-changes, *p*-values and averaged abundances were visualized in Voronoi treemaps as color gradients and cell sizes, respectively (Figures 5A–D and Supplementary Table 3). A subset of the most strongly induced proteins in the Calu-3 proteome after SARS-CoV-2 infection is listed in Table 1.

**TABLE 1:**
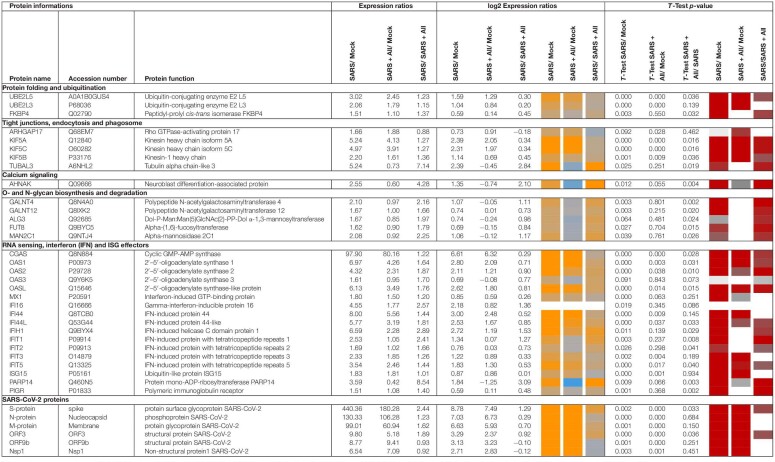
Selected most strongly induced proteins in the Calu-3 proteome after SARS-CoV-2 infection and the effect of allicin treatment p.i.

*The proteome samples of Calu-3 Mock cells (Mock), SARS-CoV-2 infected Calu-3 cells (SARS) and SARS-CoV-2 infected Calu-3 cells treated with 150 μM allicin p.i. (SARS + All) were harvested after 24 h p.i. and separated by non-reducing SDS PAGE for prefractionation. Protein fractions were tryptic in-gel digested and peptides analyzed by Orbitrap Fusion LC-MS/MS analysis as described in the “Materials and Methods”. The table lists 37 out of 207 identified proteins with >1.5-fold induction upon SARS-CoV-2 infection. These proteins are most strongly induced after SARS-CoV-2 infection, affected by allicin treatment and/or are present at high abundance in the Calu-3 proteome. The proteins were classified according to their KEGG ontologies and UniprotKB annotations. The full set of up- and downregulated proteins after SARS-CoV-2 infection is listed in Supplementary Table 3. The table includes protein names, accession numbers, protein functions, expression ratios, log2 expression ratios and *p*-values. The log2 ratios and *p*-values are visualized with a blue-orange and red-gray color code, respectively. *p*-values were calculated using an unpaired two-tailed *t*-test for two samples with unequal variance.*

**FIGURE 6:**
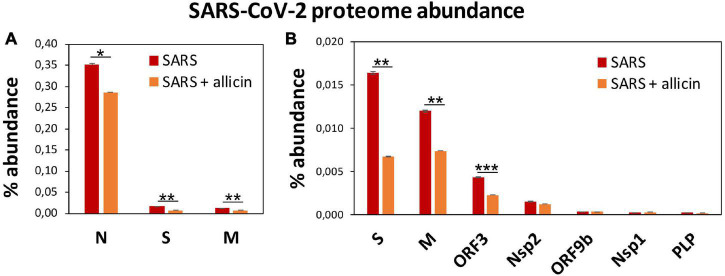
The effect of allicin on the abundance of SARS-CoV-2 proteins in the proteome of infected Calu-3 cells. **(A,B)** The abundance of the SARS-CoV-2 proteins (N, S, M, ORF3, Nsp2, ORF9b, Nsp1, and PLP) at 24 h p.i. relative to the total proteome abundance of infected Calu-3 cells was calculated in the absence (SARS) or presence of 150 μM allicin exposure p.i. (SARS + allicin). The N-protein showed a 21–29-fold higher abundance compared to the structural proteins S and M as shown in panel **(A)**. The lower abundant S, M, ORF3, Nsp2, ORF9b, Nsp1, and PLP proteins are separately displayed in panel **(B)**. The proteome results were obtained from 3–4 biological replicates with 1–3 technical replicates (Supplementary Table 3). Error bars represent the SD. *p*-values were calculated using an unpaired two-tailed *t*-test. *p < 0.05; **p < 0.01; ***p < 0.001.

The proteome after SARS-CoV-2 infection revealed altered expression of various cellular pathways, including the interferon-stimulated gene (ISG) signature, transcription, translation and protein degradation, the cytoskeleton, vesicular trafficking and tight junctions, apoptosis, signal transduction pathways as well as carbon, lipid and nucleotide metabolism (Figure 5A, Table 1, and Supplementary Table 3). In addition, the eight detected SARS-CoV-2 proteins were induced after 24 h p.i. of Calu-3 cells, with the ribonucleocapsid protein (N-protein) as one of the most abundant proteins in the proteome of infected Calu-3 cells with 0.35% of the total proteome. The N-protein was 29- and 21-fold higher expressed compared to the membrane protein (M-protein) (0.012%) and spike protein (S-protein) (0.016%), respectively (Figure 6A and Supplementary Table 3), confirming previous data with infected Vero E6 cells (Zecha et al., 2020). The viral proteins Nsp1, Nsp2, ORF3, ORF9b, and the papain-like protease PLP were low abundant, contributing from 0.00022% (PLP) to 0.0043% (ORF3) to the total proteome (Figure 6B and Supplementary Table 3), while other viral proteins were not detected.

SARS coronaviruses have been shown to enter the cell via endocytosis and direct fusion with the cell membrane (Wang et al., 2008; Ou et al., 2020). In agreement with these reports, 18 proteins involved in vesicular transport and cytoskeleton regulation, such as formation of lysosomes, phagosomes, and exosomes were 1.5–5.2-fold higher expressed after infection in the Calu-3 proteome (Figure 5A, Table 1, and Supplementary Table 3). Among these proteins are the abundant and highly induced kinesins (KIF5A/B/C), clathrin (CLTCL1), and tubulin (TUBAL3), which are microtubule-associated proteins and participate in endocytosis and traffic of viral RNA and vesicles. The 1.7-fold induced highly abundant Rho GTPase-activating protein 17 (ARHGAP17) could be involved in the repair of tight junctions, which are often damaged in COVID-19 patients (De Maio et al., 2020; Tian et al., 2020).

**FIGURE 7:**
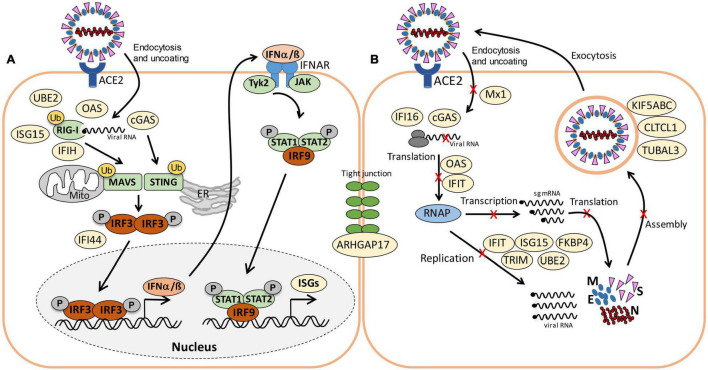
Schematic of viral RNA recognition, activation of the IFN and ISG signaling pathways **(A)** and antiviral functions of the identified ISG effectors **(B)** in this host-virus proteome study. **(A)** SARS-CoV-2 enters host cells via endocytosis. RIG-I is a cytosolic receptor to recognize viral RNA. cGAS and OAS are ISG effectors that function as RNA sensors. RIG-I, IFIH, and cGAS activate the mitochondrial antiviral-signaling protein (MAVS) and stimulator of IFN genes (STING), leading to phosphorylation of IFN responsive factors (e.g., IRF3), followed by IRF3 dimerization, translocation into the nucleus and transcriptional activation of IFN expression. IRF3 is negatively regulated by IFI44. RIG-I, MAVS, and STING can be regulated by ubiquitination (UBE2) and ISGylation (ISG15). Type-I IFN a/ß bind to the IFNAR receptor, resulting in phosphorylation of signal transducers and activators of transcription (STAT1/2) by the JAK and TYK kinases. Phosphorylated STAT1/2 form dimers and bind to IRF9, which triggers transcription of IFN-stimulated genes (ISGs) in the nucleus. **(B)** Antiviral ISG effectors affect different stages of the viral life cycle. Mx1 inhibits virus endocytosis and uncoating of the ribonucleocapsid. IFI16, OAS, and IFIT function in viral RNA degradation and block translation. IFIT, ISG15, TRIM, and UBE2 inhibit transcription, replication, translation or virus assembly. FKBP4 promotes protein folding. Kinesins (KIFA/B/C), Clathrin (CLTCL1), and TUBAL3 are involved in transport of virus vesicles. ARHGAP17 facilitates the formation or repair of tight junctions. The figure is adapted from reference (Schneider et al., 2014).

About 21 proteins of the interferon (IFN) and ISG response were strongly induced, including sensors of viral RNA, the JAK-STAT signal transduction pathway and antiviral effectors that interfere with the viral life cycle (Figure 5A, Table 1, and Supplementary Table 3) (Schneider et al., 2014). For a better understanding, the RNA sensing receptors, IFN and ISG signaling cascades and the previously described antiviral functions of the ISG effectors are displayed in a schematic (Figures 7A,B). The cyclic GMP-AMP (cGAMP) synthase (cGAS) was most strongly 98-fold upregulated upon infection, acting as sensor of viral RNA (Table 1, Supplementary Table 3, and Figure 5A) (Schneider et al., 2014). cGAMP activates the stimulator of interferon genes (STING) (Figure 7A). The 2′-5′-oligoadenylate synthases (OAS1-3, OASL) were 1.6–7-fold induced upon infection to produce 2′-5′-adenylic acid as second messenger and activator of RNaseL for viral RNA degradation. The IFN-induced helicase C-domain-containing protein (IFIH) was 6.5–fold upregulated, which activates the mitochondrial antiviral signaling protein (MAVS) to induce the IFN response. Other IFN-induced effector proteins with tetratricopeptide repeats (IFIT1-3, IFIT5) were 1.6–3.5-fold induced after infection and function in RNA degradation and inhibition of translation. Further effectors are the Interferon-induced myxoma resistance protein 1 (MX1) and the Polymeric immunoglobulin receptor (PIGR), which represented 0.05 and 0.1% of the total proteome abundance and were 1.8 and 1.5-fold induced, respectively. MX1 is a dynamin-like GTPase, which forms ring-like structures and traps incoming ribonucleocapsids, thereby blocking uncoating and vesicular trafficking to direct them for degradation (Figure 7B) (Schneider et al., 2014). MX1 was also reported to be up-regulated in COVID-19 patients (Bizzotto et al., 2020).

Furthermore, the abundant cytokine IL18, the IL-1 receptor antagonist protein (IL1RN), the macrophage immunometabolism regulator MACIR and the Alpha-2-macroglobulin (A2M) were ∼0.6-fold lower expressed in infected cells. MACIR is implicated in the regulation of macrophages and autoimmune diseases (McGauran et al., 2020).

An important role in signal transduction and regulation of the antiviral response plays the abundant ISG15 effector, which was 1.8-fold induced after SARS-CoV-2 infection in the proteome. ISG15 functions amongst others as Ubiquitin-like modifier in ISGylation of RIG-I and IRF-3, which are targeted for degradation or activated to regulate IFN and ISG production (Figure 7A) (Masucci, 2020). Widespread ISGylation of newly synthesized viral proteins is proposed to inhibit viral replication and translation (Durfee et al., 2010).

Additionally, post-translational modification by polyubiquitination of host signaling factors, such as RIG-I, STING and MAVS is important for regulation of the IFN response upon SARS-CoV-2 infection (Figure 7A). Thus, several ubiquitin-conjugating E2 enzymes (UBE2L3 and UBE2L5), the E3 ubiquitin ligases (TRIM21, TRIM38, and ARIH2) and the ubiquitin specific protease or deconjugases (USP13) are 1.5–3.2-fold induced in the infected cells, while other E2, E3 enzymes and deconjugases (e.g., UBE2D2/3, RNF214, USP4, USP47, and USP48) are 0.2–0.62-fold lower expressed (Table 1, Supplementary Table 3, and Figure 5A). Since host and viral targets of ubiquitination and ISGylation are often directed to degradation, components of the proteasome, proteases, protein folding factors, and chaperones are 1.5–1.8-fold upregulated. The folding factors include the highly abundant peptidyl-prolyl *cis*-*trans* isomerase FKBP4, which functions as immunophilin and co-chaperone to interact with HSP90.

Apart from protein modification, the virus relies on protein synthesis and translation by the host machinery for its successful replication and infectivity. Accordingly, 24 proteins involved in translation were 1.5–2.8-fold upregulated under SARS-CoV-2 infection, including the translation factor EIF2B1, ribosomal proteins (RPL26, MRPS30, RRP8, PDCD11, and MRPL4), RNA helicases (DDX55 and DDX56), RNAses (POP1 and XRN1) and other regulatory factors, such as phosphatases (PPP1CC, PPP1CA, and PPP2R5A) (Supplementary Table 3 and Figure 5A).

In addition, 16 proteins involved in transcription and the spliceosome were upregulated in infected Calu-3 cells, including the pre-mRNA splicing factors Slu7, PRPF40B, SCAF11, and the U1 small nuclear ribonucleoprotein C (SNRPC), which were 1.6–1.8-fold higher expressed. The transcription factors GABPA, ZNF579, SP110, and TSC22D2 were also induced after infection. However, the majority of differentially expressed proteins involved in transcription (48) and translation (30) were repressed after SARS-CoV-2 infection, including the highly abundant proteins DIDO1, SUB1, FUBP1, TCEA1, BOP1, RPS24, and RPS15 (Supplementary Table 3 and Figure 5A).

Moreover, virus replication and proliferation inside host cells requires reprogramming of the host metabolism, which was evident by the upregulation of 34 proteins and downregulation of 43 proteins involved mainly in lipid, energy, glycan, and nucleotide metabolism (Table 1, Supplementary Table 3, and Figure 5A). The induced proteins might function in the biosynthesis of the building blocks for viral phospholipid membranes, glycosylation of surface proteins and viral RNA genomes. Since the nucleotide pool is essential for coronavirus replication (Bojkova et al., 2020), some purine and pyrimidine biosynthesis proteins were 1.7–2.3-fold induced (NT5C2, UPP1, and PPAT), while others were 0.5-0.65-fold repressed (CMPK1, AK6, and ENPP4) (Supplementary Table 3 and Figure 5A).

Furthermore, expression of several signaling pathways, including JAK-STAT, MAPK, Wnt, Ras, and Rap1 signaling were affected by SARS-CoV-2 infection. The JAK-STAT pathway senses and transduces IFN-signals via a phosphorylation cascade to activate ISG expression (Figure 7A). Thus, STAT2, N-myc interactor NMI and the RIG-I receptor were 1.6–1.8-fold induced upon infection (Supplementary Table 3 and Figure 5A). Proteins of the MAPK signaling pathways were activated in response to infections with SARS-CoV (Bouhaddou et al., 2020) and 1.6–1.8-fold induced in the proteome of SARS-CoV-2 infected cells. Proteins of the PI3K/Akt signaling pathway were 2.3–2.6-fold upregulated in infected cells, controlling apoptosis of host cells for successful viral replication. The highly abundant neuroblast differentiation-associated protein AHNAK was 2.6-fold induced after SARS-CoV-2 infection. AHNAK is required for calcium signaling and might regulate the immune response (Matza et al., 2009). Proteins of the Ras-signaling pathway were 0.5–0.64-fold downregulated upon virus infection, including three Rac GTPases Rac1-3 that are implicated in the regulation of cell morphology, migration and invasion, by transducing signals from cell surface receptors to the actin and microtubule cytoskeletons (Wheeler et al., 2006). Similarly, other proteins involved in the cytoskeleton organization were 0.3–0.66-fold lower expressed, indicating re-organization of the cytoskeleton for transport of virus particles.

### Allicin Leads to a Decreased Antiviral Interferon Response in the Proteome of Infected Calu-3 Cells

Next, we investigated the effect of allicin on the proteome changes upon SARS-CoV-2 infection. Quantification of the 8 viral proteins in infected Calu-3 cells after allicin treatment revealed a significantly 18–59% decreased abundance of the structural proteins N, M, and S and ORF3, supporting the antiviral effect of allicin in the proteome (Figures 6A,B).

Allicin treatment resulted in a diminished IFN-response in infected cells, since expression of innate immune receptors and ISG effectors of the JAK-STAT signaling pathways were decreased, including FKBP4, PIGR, MX1, cGAS, OAS1-3, and IFIT1-3 (Table 1, Supplementary Table 3, and Figures 5B,C). In addition, proteins involved in ubiquitination (UBE2L3/5) and the JAK-STAT, MAPK, PI3K/Akt, and Ras signaling pathways showed lower expression changes after allicin treatment. The abundant calcium-signaling protein AHNAK was repressed after allicin exposure, while it was induced in infected cells. Allicin resulted in decreased expression of kinesins KIFA/B/C, clathrin CLTCL1 and tubulin TUBAL3, indicating reduced endocytosis and traffic of vesicles. Moreover, prothymosin alpha (PTMA) was 2.6-fold upregulated after allicin exposure. PTMA showed antiviral activity to inhibit replication of human deficiency virus type-1 (Mosoian et al., 2006; Teixeira et al., 2015), indicating that PTMA induction by allicin might contribute to the antiviral effect against SARS-CoV-2.

Similarly, the expression of proteins involved in transcription, spliceosome and translation was reversed to the levels of uninfected (Mock) cells after allicin exposure of SARS-CoV-2 infected cells, including the abundant proteins DIDO1, SUB1, FUBP1, TCEA1, BOP1, RPS24 and RPS15. Finally, expression of metabolic enzymes involved in glycan, nucleotide, and lipid metabolism was restored by allicin, including GALNT4/12, ALG3, FUT8, MAN2C1, CMPK1, and TECR (Table 1, Supplementary Table 3, and Figures 5B,C). Overall, allicin showed antiviral effects in the host proteome as revealed by the diminished IFN-dependent antiviral response and the effects on signal transduction, transcription, translation, and metabolism.

## Discussion

Garlic organosulfur compounds showed antiviral activity against several enveloped viruses, including herpes simplex, parainfluenza, vaccinia, and rhinovirus (Weber et al., 1992; Rouf et al., 2020). These virucidal effects of garlic compounds were proposed to depend on the disruption of the viral envelope and inhibition of viral replication (Weber et al., 1992; Rouf et al., 2020).

In this work, we explored the antiviral effect of allicin on SARS-CoV-2 infected Vero E6 and Calu-3 cells. By determining >60–70% decreased levels of viral RNA and infectious viral particles, the antiviral effect of biocompatible allicin doses against SARS-CoV-2 was demonstrated in both cell lines. However, Calu-3 cells showed a greater allicin tolerance compared to the more sensitive Vero E6 cells. Different cell lines were previously shown to vary in their allicin susceptibilities, which correlated with their intracellular GSH levels (Gruhlke et al., 2016, 2019). Allicin leads to *S*-thioallylation of GSH and the formation of *S*-allylmercaptoglutathione (GSSA), which is accompanied by GSH depletion and an oxidative shift in the GSH redox potential (Gruhlke et al., 2010, 2019; Müller et al., 2016). The measurement of GSH levels confirmed that Calu-3 cells have 4.2-fold higher GSH levels compared to the allicin-sensitive Vero E6 cells.

Since Vero E6 cells are more sensitive toward allicin compared to Calu-3 cells, we first analyzed the effect of low levels of 50 μM allicin on SARS-CoV-2 infected Vero E6 cells using pre- and post-infection treatments of host cells or pre-exposure of the virus before infection. Interestingly, treatment of Vero E6 cells with 50 μM allicin showed only antiviral effects if applied after infection with SARS-CoV-2. The number of infectious viral particles was not affected if virus or Vero E6 host cells were pretreated with 50 μM allicin for 30 min before infection. This missing antiviral effect of allicin in the pre-treatment experiments might be explained by the washing step of Vero E6 cells after the 1 h infection, leading to the removal of the remaining allicin from the cells. During post-infection treatment, allicin remained in the cell culture until sample harvesting. These results suggest that allicin might disrupt early steps of viral replication in Vero E6 cells, which requires further investigation. Further experiments revealed the significant reduction in viral plaques and RNA copies in both SARS-CoV-2 infected Vero E6 and Calu-3 cell lines after exposure to sub-lethal doses allicin p.i.

To better understand the effect of allicin, we investigated the proteome changes of Calu-3 cells upon SARS-CoV-2 infection and the impact of 150 μM allicin on the host-virus proteome. In agreement with previous proteome studies, SARS-CoV-2 reprograms major host pathways, including signaling pathways, transcription, splicing, translation, protein modification and folding, lipid, glycan, and nucleotide metabolism (Table 1, Supplementary Table 3, and Figures 5A, 7A,B) (Bojkova et al., 2020; Bouhaddou et al., 2020; Zecha et al., 2020). The ribonucleocapsid protein was the most abundant viral protein in the infected cell, indicating that a large portion of the translation capacity goes to the N-protein for package of the viral RNA genome.

In addition, our proteome data highlight the importance of the IFN pathway and ISG effectors to prevent virus replication by interacting with various stages of the viral life cycle. Antiviral ISG effectors were among the most highly induced and abundant proteins in the infected host cells, such as MX1, cGAS, OAS1-3, IFIT1-3, ISG15, FKBP4, PIGR, and UBE2L3/5, which function in sensing and degradation of viral RNA, inhibition of ribonucleocapsid uncoating, translation and promote the innate immune response (Table 1, Supplementary Table 3, and Figures 5A, 7A,B). Apart from IFN signaling, proteins involved in motility, tight junction and membrane trafficking are highly induced host proteins, supporting the importance of vesicular transport for virus endocytosis and exocytosis. Thus, our proteomics studies reflect all described host pathways known to be altered after viral infections, suggesting new host targets for SARS-CoV-2 interventions.

At the same time, the proteomic profiling gave the opportunity to monitor the responses of infected Calu-3 cells after allicin treatment. The proteome results of allicin-treated infected host cells revealed an 18–59% reduced abundance of the structural proteins N, M, S, and ORF3. Several expression changes dedicated to virus proliferation are reversed to Mock levels in allicin-treated cells. Allicin affected virus-responsive expression of JAK-STAT, MAPK, PI3K/Akt, and Ras signaling pathways, IFN and ISG effectors, transcription, splicing, translation, ubiquitination, vesicular transport, tight junctions as well as glycan, lipid, and nucleotide metabolism. Thus, our results confirm the antiviral effect of allicin in host cells in agreement with the infection assays.

The mode of action of allicin involves *S*-thioallylation of proteins and low molecular weight thiols in bacteria and human Jurkat cells, which was observed already 10–30 min after allicin treatment (Gruhlke et al., 2019; Loi et al., 2019). The majority of *S*-thioallylated Jurkat proteins were abundant cellular proteins, involved in the cytoskeleton, translation and protein folding, although also low abundant redox-sensitive transcription factors, such as MgrA, SarZ, OhrR, HypR, and YodB were targets for allicin modification in *S. aureus* and *B. subtilis* cells (Chi et al., 2019; Gruhlke et al., 2019; Loi et al., 2019). In this study, we did not detect *S*-thioallylated Cys peptides in SARS-CoV-2 infected Calu-3 cells after 24 h of allicin exposure using label-free proteomics and MS/MS spectrum verification. In addition, no viral *S*-thioallylated Cys peptides were identified, although the spike protein is a Cys-rich glycoprotein exposed on the surface of the virus envelope (Ou et al., 2020). Given the long duration of allicin treatment, this is not surprising since cells have the capacity to reduce allicin and the majority of *S*-thioallylations within 24 h. In human Jurkat cells, allicin led to a rapid depletion of the cellular GSH pool within 10 min (Gruhlke et al., 2019). Efficient allicin detoxification and removal of *S*-thioallylations were confirmed in yeast and bacterial cells, as supported by fast recovery of growth after a short allicin-induced lag phase (Gruhlke et al., 2010, 2019; Müller et al., 2016; Loi et al., 2019). In *S. aureus*, allicin can be reduced by the disulfide reductase MerA, while the bacillithiol disulfide reductase YpdA enables the recycling of *S*-allylmercaptobacillithiol, formed in the reaction of BSH with allicin (Loi et al., 2019). Furthermore, YpdA was shown to function in the Brx/BSH/YpdA pathway in regeneration of *S*-thioallylated proteins (Loi et al., 2019). Similarly, Calu-3 cells should have reduced GSSA via the glutathione disulfide reductase and *S*-thioallylated proteins by the glutaredoxin/GSH/glutathione disulfide reductase system within 24 h.

Finally, the question arises about the antiviral mechanism of allicin on SARS-CoV-2 infected Calu-3 cells during host-pathogen interactions. Garlic organosulfur compounds were shown to exert their immunomodulatory activity via inhibition of the transcription factor NF-κB, leading to decreased levels of pro-inflammatory cytokines, such as TNF-α, IL-1β, IL-6, MCP-1, and IL-12 (Arreola et al., 2015). Allicin further stimulates the release of Zn^2+^ from proteins in murine EL-4 T-cells, possibly due to *S*-thioallylation of Zn^2+^ coordinating Cys thiolates (Gruhlke et al., 2019). Thus, the immunomodulatory effect of allicin on cytokine secretion in cell cultures could be mediated by elevated Zn^2+^ levels due to inactivation of host proteins by *S*-thioallylation.

On the other hand, allicin could also target Cys-containing virus proteins, such as the Cys-rich spike glycoprotein, the viral RNA-dependent RNA polymerase RdRp (Nsp12), the main protease M^*pro*^ (also termed as 3C-like protease) and the papain-like protease PLP. M^*pro*^ and PLP are both involved in proteolytic processing of the large pp1a and pp1ab polyproteins to produce functional polypeptides, which assemble into the replicase-transcriptase complex (Jin et al., 2020; Zhang et al., 2020; Amin et al., 2021). Since M^*pro*^ and RdRp are important for viral replication and transcription, these could be antiviral drug targets. M^*pro*^ has a catalytic active site motif consisting of His41 and Cys145 residues (Jin et al., 2020; Zhang et al., 2020; Amin et al., 2021). Several *in silico* docking studies with allicin revealed the formation of *S*-thioallylations at Cys145, Cys85 and Cys156 of M^*pro*^ and of Cys622 of RdRp, indicating the potential of allicin to attenuate SARS-CoV-2 replication (Bastikar et al., 2020; Shekh et al., 2020). Further docking studies with the garlic compounds alliin and ajoene revealed strong ligand-protein binding stabilities and many interactions at the M^*pro*^ active site (Bastikar et al., 2020; Cheng and Li, 2020). In total, 17 garlic organosulfur compounds, accounting for 99.4% of substances found in garlic oil, showed interactions with ACE2 receptor and M^*pro*^ *in silico*, including the diallyl di- and trisulfides with promising docking scores (Thuy et al., 2020). Furthermore, elevated Zn^2+^ levels released from host proteins inhibited the RdRp of SARS coronavirus (te Velthuis et al., 2010) and the host ACE2 enzyme (Polak and Speth, 2021), indicating that allicin could target either host and viral proteins directly via *S*-thioallylation or via elevated Zn^2+^ levels to exert immunomodulatory and antiviral effects.

Thus, allicin might inhibit SARS-CoV-2 infection at different stages of the viral life cycle, preventing receptor binding, replication or transcription by *S*-thioallylation of host or viral proteins. However, in our host-virus proteome we could not identify the M^*pro*^ or RdRp proteins, indicating that these are low abundant proteins. Thus, possible *S*-thioallylations of M^*pro*^ and RdRp will be difficult to verify upon allicin exposure of infected host cells *in vivo*. Nevertheless, these *in silico* docking studies highlight the potential of garlic organosulfur compounds as inhibitors of viral Cys proteins, which could be further developed as possible future COVID-19 therapeutics.

Taken together, our results demonstrate that allicin shows antiviral and immunomodulatory activity in SARS-CoV-2 infected Vero E6 and Calu-3 cell cultures, supported on the proteome level by the decreased antiviral interferon response. However, allicin is unstable and quickly decomposes to polysulfanes, ajoene, and other sulfur compounds during heating (Block, 2010; Borlinghaus et al., 2021). The half-life of allicin is 30–40 days in water at 23°C, but decreases in garlic extracts with increased concentrations (Koch and Lawson, 1996). In the acidic stomach, the majority of allicin is degraded to 2-propenethiol and allyl methyl sulfide, which are excreted (Block, 2010; Borlinghaus et al., 2021). In the blood, the effective dose of allicin is reduced by its reaction with GSH (Block, 2010; Borlinghaus et al., 2021). In our experiments, the viral load was only reduced by 60–70% after allicin treatment in Calu-3 cells, which is below 1-log scale and would not satisfy the desired antiviral effect required for therapeutics to enter pre-clinical trials. Future drug research should be directed to exploit the thiol-reactive activity of allicin derivatives with reduced toxicity, increased stability and higher antiviral activity as antiviral lead compounds.

## Data Availability Statement

The mass spectrometry data have been deposited to the ProteomeXchange Consortium via the PRIDE partner repository (Perez-Riverol et al., 2019; Deutsch et al., 2020) with the dataset identifier PXD024375.

## Author Contributions

KM performed the infection experiments and analyzed the data. VF and LA measured and analyzed the proteomic data. JB constructed the Voronoi treemaps. MG and AS synthesized allicin. DN supervised the infection experiments and gave critical advise. VF and HA conducted the study and wrote the initial manuscript. All authors contributed to the final manuscript.

## Author Disclaimer

Crushed garlic acts as strong irritant and activates the transient receptor potential channel (TRPA1) in pain-sensing neurons, resulting in pain, inflammation and neurotoxic effects (Bautista et al., 2005). Garlic can be highly toxic to human cells and caused severe garlic burns by its direct exposure to the skin or mucous membranes of the respiratory tract (Bautista et al., 2005; Al-Qattan, 2009; Vargo et al., 2017; Hitl et al., 2021; Muniz et al., 2021). Consequently, any ingestion, inhalation or introduction of fresh garlic can clearly be harmful and hazardous for patients, causing even more severe damage of the lung or the skin. This work does in no way suggest the use of garlic for self-medication of respiratory tract infections, including COVID-19. The authors explicitly warn against any form of self-medication with garlic or garlic compounds.

## Conflict of Interest

The authors declare that the research was conducted in the absence of any commercial or financial relationships that could be construed as a potential conflict of interest.

## Publisher’s Note

All claims expressed in this article are solely those of the authors and do not necessarily represent those of their affiliated organizations, or those of the publisher, the editors and the reviewers. Any product that may be evaluated in this article, or claim that may be made by its manufacturer, is not guaranteed or endorsed by the publisher.
